# Stress Level of General Dentists and Pedodontists in Dental Treatment of Pediatric Patients: Stress Level of Dentists in Dental Treatment of Pediatric Patients

**DOI:** 10.31661/gmj.v13iSP1.3620

**Published:** 2024-12-31

**Authors:** Razie Meshki, Niloofar Salehian, Sahar Tehrani

**Affiliations:** ^1^ School of Dentistry, Ahvaz Jundishapur University of Medical Sciences, Ahvaz, Iran; ^2^ Department of Pediatric Dentistry, School of Dentistry Ahvaz Jundishapur University of Medical Sciences, Ahvaz, Iran

**Keywords:** Stress, Psychological, Pediatric Dentistry, Dentists, Local Anesthesia

## Abstract

**Background:**

This study aimed to assess the stress level of general dentists and pedodontists in dental treatment of pediatric patients.

**Materials and Methods:**

This cross-sectional study was conducted on 101 general dentists and 93 pedodontists who were selected from different provinces of Iran by stratified random sampling. A researcher-designed questionnaire was used for data collection, which included four sections of demographics, trait stress, functional stress, and attitude towards the efficacy of behavioral control measures for pediatric dental patients. Data were analyzed by ANOVA, Bonferroni test, Chi-square test, and independent t-test.

**Results:**

The mean age of the participants was 37.93 years. The participants reported the highest level of stress during administration of an inferior alveolar nerve (IAN) Block for anxious children, and the lowest stress during cavity preparation for an amalgam restoration of a mandibular tooth. In all procedures, pedodontists reported lower stress level than general dentists; except for the Distraction technique, Modeling technique, presence of child’s parents, and examination of child (P0.05). The stress level of males was lower than females during administration of an IAN Block (P0.05). The highest efficacy score was given to the Tell-Show-Do technique and the lowest score was allocated to the hand-over-mouth technique. The attitude of pedodontists was more positive than general dentists regarding the efficacy of behavioral control techniques.

**Conclusion:**

The results showed that administration of an IAN block for an anxious child was the most stressful procedure for both general dentists and pedodontists. The stress level of pedodontists was generally lower than that of general dentists in all procedures.

## Introduction

Stress is defined as the human response to any demand, change or perceived threat [1]. Although both healthy and harmful forms of stress are present, in psychology, the term "stress" mainly refers to harmful stress, which impairs correct thinking and learning, and may even lead to physical problems such as cardiovascular complications (e.g., tachycardia), gastrointestinal disorders, insomnia, headache, and excessive sweating [1]. It appears that dentists are at a higher risk of perceiving stress and developing anxiety disorders, including generalized anxiety disorder (GAD), due to the demanding nature of their profession. A study by Queirolo et al. (2023) found that a significant number of young dentists experienced moderate levels of GAD during a 24-hour period of a working day [2]. Dentists are prone to anxiety disorders and clinical depression due to the nature of their clinical practice and high expectations [3]. In a study in Saudi Arbia, 77.14% of dentists had symptoms of marked to severe anxiety [4]. Pediatric dentists, in particular, may be at greater risk for occupational burnout and/or depression due to chronic stress associated with providing pediatric dental care and the increasing prevalence of females in the workforce [5]. Paediatric dentists often experience high levels of stress when providing dental care to children, particularly when sedation is involved [6][7][8].

Research has shown that the stress levels of paediatric dentists can be influenced by various factors, including the child’s behaviour during treatment, the dentist’s level of experience, and the type of sedation [6][8]. For instance, a study found that paediatric dentists reported higher levels of stress when treating children under deep sedation compared to clinical and general anesthesia [8]. Additionally, the child’s behaviour during treatment, such as struggling or non-cooperation, can also contribute to increased stress levels in paediatric dentists [6]. Furthermore, studies have suggested that experience is an important factor in reducing stress, with more experienced paediatric dentists reporting lower levels of stress [9]. Crying, shivering, anger, and avoidant behaviors of pediatric dental patients often irritate the dental clinicians since they need to put more energy to manage such patients [10]. All dental procedures such as anesthetic injection, rubber dam placement, restoration, or extraction of teeth can be stressful for dental clinicians. Pedodontists need to have a different approach for behavioral control of children and also to manage their own stress level [11]. Thus, this study aimed to assess the stress level of general dentists and pedodontists in dental treatment of pediatric patients.

## Materials and Methods

This cross-sectional study was conducted on general dentists and pedodontists in 2022 in Iran, including faculty members and practitioners working in both private and public clinics. The participants were recruited through a convenience random sampling method, where a total of 123 general dentists and 123 pedodontists were selected from different provinces of Iran by stratified random sampling. The participants were invited to participate in the study through SMS and email invitations, using contact information obtained from the Iranian Medical Council. The participants’ phone numbers were retrieved from the Iranian Medical Council database, and they were contacted via SMS and email to request their participation in the study, with a clear explanation of the study objectives, procedures, and confidentiality measures. Of a total of 246 participants, 194 filled out and returned the questionnaires including 101 general dentists and 93 pedodontists. The response rate was 78.86% among those who were approached.

### 1. Eligibility Criteria

The inclusion criterion was general dentists and pedodontists who were willing to participate in the study. Those who did not fill out over 20% of the questionnaire were excluded.

### 2. Sample Size

A priori sample size calculation was performed to determine the required number of participants to detect significant differences in stress levels between high-experience and low-experience dentists. Using a two-tailed test with a moderate effect size (Cohen’s d=0.5), a significance level of 0.05, and a power of 0.80, we estimated the required sample size using G*Power software (latest ver. 3.1.9.7; Heinrich-Heine-Universität Düsseldorf, Düsseldorf, Germany). Based on the proportions of dentists with severe stress levels in each experience group based on the study of Azher et al. [12] (3.8% for ≤ 5 years and 12.1% for>5 years), the sample size calculation yielded a required sample size of 123 dentists per group to detect a significant difference in stress levels.

### 3. Selection of the Participants

#### 3.1. Data Collection

A researcher-designed questionnaire was used to assess the stress level of general dentists and pedodontists. This questionnaire was designed according to previous studies [10][13] after thorough evaluation of the questions, and had four parts of demographics, trait stress, functional stress, and attitude towards behavioral control measures for pediatric dental patients. The validity of the questionnaire was ensured according to the opinion of the experts after making the necessary changes. Its reliability was also ensured by calculation of the Cronbach’s alpha coefficient. The calculated Cronbach’s alpha coefficient was 0.845 for final version in assessing it in a pilot study with 20 participants.

Another questionnaire was used to assess the additional data on participants’ demographic characteristics, including marital status, number of children (if married), provision of dental treatment for children under 12 years, conduction of dental treatment in presence of parents, and the parameter causing stress during dental treatments for children (child, parents, or dentist himself).

### 3.2. Scoring

Trait stress: This section included 10 questions with 6-point Likert scale answer choices of never (0), very low (1), low (2), high (3), very high (4), and always (5). Higher scores indicated higher stress level. The scoring was reverse for questions 1-3: never (5), very low (4), low (3), high (2), very high (1), and always (0). The total score of this domain could range from 0 to 40, and higher scores indicated higher level of trait stress. Thus, the trait stress was dichotomized as low level (0-20) and high level (21-40); score 20 indicated average trait stress.

Functional stress: This section indicated the stress level of dental clinicians when performing different dental procedures. It included 14 questions assessing 7 commonly performed procedures in the maxilla and mandible in a parallel design. The questions had 6-point Likert scale answer choices of never (0), very low (1), low (2), high (3), very high (4), and always (5). Higher scores indicated higher stress level. The total score of this domain could range from 0 to 70, and higher scores indicated higher level of functional stress. Thus, the functional stress was dichotomized as low level (0-35) and high level (36-70); score 35 indicated average functional stress.

Total stress: This section included the sum of trait and functional stress scores and its total score could range from 0 to 120, and higher scores indicated higher level of total stress. Total stress score <60 indicated low stress and >60 indicated high stress level; score 60 indicated average total stress. A three-level classification was also considered and scores 0-40 indicated low, 41-80 indicated moderate, and 81-120 indicated high total stress level.

Attitude: The participants were asked to express their attitude towards the efficacy of behavioral control measures for pediatric dental patients. This section had 7 questions with 6-point Likert scale answer choices of never (0), very low (1), low (2), high (3), very high (4), and always (5). Higher scores indicated higher efficacy of the respective technique for behavioral control of children according to the opinion of the participants. The total attitude score could range from 0 to 35; higher scores indicated a more positive attitude towards optimal efficacy of the measures. The average score was 17.5. Scores<17.5 indicated that the participant believed that the behavioral control measures have a low or very low efficacy while scores >17.5 indicated that the participant believed that the behavioral control measures are highly or very highly effective.

### Statistical Analysis

Independent t-test was used to compare the stress level between males and females. It was also used for other dichotomous variables. ANOVA was applied to compare the stress level of general dentists and pedodontists followed by the Bonferroni post-hoc test. The Chi-square test was applied to compare the stress level among two groups. Level of statistical significance was set at 0.05.

## Results

**Table:** Table1. Demographic Variables of the Participants

**Variable**	**Category**	**Number**	**Percentage**
Gender	**Female**	161	83
	**Male**	33	17
Marital status	**Married**	131	67.5
	**Single**	63	32.5
	**No child**	51	38.9
Number of children (if married)	**1**	33	25.2
	**2**	43	32.8
	**3**	4	3.1
Education	**Pedodontist**	93	47.9
	**General dentist**	101	52.1
Provision of dental treatment for children under 12 years	**Yes**	183	94.3
	**No**	11	5.7
Conduction of dental treatment in presence of parents	**Yes**	158	81.4
	**No**	36	18.6
	**Child**	50	25.8
Parameter causing stress during dental treatments for children	**Parents**	139	71.6
	**Dentist himself**	5	2.6

**Table:** Table2. Comparison of Pedodontists and General Dentists Regarding Provision of Dental Care for Children under 12 years, Conduction of Dental Treatment in Presence of Parents, and the Main Factor Causing Stress in Clinicians

**Variable**			**Academic education**			
	**Pedodontists**		**General dentists**		P-value*	
	**n**	**%**	**n**	**%**	
Provision of dental care for children under 12 years	**Yes**	100	93	90	89.1	<0.001*
	**No**	0	0	11	10.9	
Conduction of dental treatment in presence of parents	**Yes**	91.4	85	73	72.3	<0.001*
	**No**	8.6	8	28	27.7	
	**Child**	22.6	21	29	28.7	0.56
Main factor causing stress in clinicians	**Parents**	74.2	69	70	69.3	
	**Dentist himself**	3.2	3	2	2	

^*^ Significant difference

**Table:** Table3. Frequency Distribution of Responses to Questions in the Trait Stress Domain of the Questionnaire

**Never**	**Very low**	**Low**	**High**	**Very high**	**Always**	**General dentists**	**Pedodontists**	**P value**
**n(%)**	**n(%)**	**n(%)**	**n(%)**	**n(%)**	**n(%)**	**Mean± SD**	**Mean± SD**	
**I prefer pediatric dental patients if I had a choice **								
62(31.96%)	27(13.92%)	37(19.07%)	36(18.56%)	21(10.82%)	11(5.67%)	2.12±1.34	0.688±0.96	<0.001*
**I am capable of managing pediatric dental patients similar to adult patients **								
36(18.56%)	57(29.38%)	63(32.47%)	30(15.46%)	4(2.06%)	4(2.06%)	2.13±1.05	1.01±0.92	<0.001*
**I have self-confidence in management of children on dental unit. **								
47(24.23%)	56(28.87%)	79(40.72%)	11(5.67%)	0(0%)	1(0.52%)	1.67±0.92	0.89±0.75	<0.001*
**I have stress during clinical dental examination of a child **								
75(38.66%)	71(36.6%)	47(24.23%)	0(0%)	1(0.52%)	0(0%)	0.97±0.88	0.76±0.71	0.077
**I have stress during clinical dental examination of an anxious child **								
49(25.26%)	66(34.02%)	64(32.99%)	13(6.7%)	2(1.03%)	0(0%)	1.44±1.02	1.02±0.79	0.001*
**I have stress during conduction of dental treatments for children in presence of their parents **								
32(16.49%)	50(25.77%)	76(39.18%)	31(15.98%)	4(2.06%)	1(0.52%)	1.71±1.05	1.53±1.01	0.24
**During dental treatment of children, concerns about the possible adverse behaviors of the child increase my stress level **								
23(11.86%)	56(28.87%)	80(41.24%)	34(17.53%)	0(0%)	1(0.52%)	1.89±0.92	1.41±0.88	<0.001*
**I am stressed out during and even after completion of dental treatment of children **								
29(14.95%)	84(43.3%)	75(38.66%)	4(2.06%)	1(0.52%)	1(0.52%)	1.46±0.81	1.15±0.76	0.006*

^*^ Statistically significant

**Table:** Table4. Frequency Distribution of Responses of the Two Groups to Questions in the Functional Stress Domain of the Questionnaire

	Never	Very low	Low	High	Very high	Always	General dentists	Pedodontists	P value
	**n(%)**	**n(%)**	**n(%)**	**n(%)**	**n(%)**	**n(%)**	**Mean± std. deviation**	**Mean± std. deviation **
				Maxillary anesthesia injection				
40(20.62%)	52(26.8%)	62(31.96%)	37(19.07%)	3(1.55%)	0(0%)	1.79±1.07	0.95±0.84	0.001*
				Cavity preparation for a mandibular amalgam restoration				
57(29.38%)	66(34.02%)	53(27.32%)	18(9.28%)	0(0%)	0(0%)	1.35±1.01	0.61±0.69	0.003*
				Cavity preparation for a maxillary amalgam restoration				
81(41.75%)	67(34.54%)	40(20.62%)	6(3.09%)	0(0%)	0(0%)	1.06±0.93	0.72±0.77	<0.001*
				Crown preparation for a mandibular tooth				
76(39.18%)	65(33.51%)	37(19.07%)	16(8.25%)	0(0%)	0(0%)	1.18±1.05	0.73±0.82	0.001*
				Crown preparation for a maxillary tooth				
71(36.6%)	60(30.93%)	47(24.23%)	13(6.7%)	2(1.03%)	1(0.52%)	1.36±1.1	0.87±0.89	<0.001*
				Pulpotomy of a primary mandibular tooth				
63(32.47%)	70(36.08%)	38(19.59%)	20(10.31%)	2(1.03%)	1(0.52%)	1.36±1.12	0.79±0.81	0.001*
				Pulpotomy of a primary maxillary tooth				
70(36.08%)	62(31.96%)	46(23.71%)	16(8.25%)	0(0%)	0(0%)	1.26±1.03	0.79±0.77	0.001*
				Pulpectomy of a primary mandibular tooth				
	71(36.6%)	64(32.99%)	39(20.1%)	20(10.31%)	0(0%)	0(0%)	1.26±1.11	1.04±0.91	0.001*
				Pulpectomy of a primary maxillary tooth				
	61(31.44%)	52(26.8%)	55(28.35%)	23(11.86%)	3(1.55%)	0(0%)	1.44±1.17	1.11±0.9	0.009*
				Extraction of a primary mandibular tooth				
	59(30.41%)	50(25.77%)	54(27.84%)	27(13.92%)	3(1.55%)	1(0.52%)	1.5±1.26	0.95±0.85	0.015*
				Extraction of a primary maxillary tooth				
	57(29.38%)	58(29.9%)	62(31.96%)	14(7.22%)	3(1.55%)	0(0%)	1.45±1.06	1.12±0.94	<0.001*
				I have stress during mandibular anesthetic injection for a child				
	53(27.32%)	57(29.38%)	62(31.96%)	19(9.79%)	3(1.55%)	0(0%)	1.43±1.07	1.49±0.93	0.037*

^*^ Statistically significant

**Table:** Table5. Frequency Distribution of Responses of the Two Groups to Questions in the Attitude Domain of the Questionnaire

Never	Very low	Low	High	Very high	Always	General dentists	Pedodontists	P value
**n(%)**	**n(%)**	**n(%)**	**n(%)**	**n(%)**	**n(%)**	**Mean± std. deviation**	**Mean± std. deviation**	
**Behavioral control measures can effectively manage dental stress in children. **			
	0(0%)	3(1.55%)	8(4.12%)	66(34.02%)	75(38.66%)	42(21.65%)	3.61±0.84	3.89±0.92	0.030*
**Tell-show-do technique **
	0(0%)	0(0%)	6(3.09%)	62(31.96%)	83(42.78%)	43(22.16%)	3.65±0.82	4.04±0.72	0.001*
**Positive reinforcement technique **							
	0(0%)	0(0%)	10(5.15%)	52(26.8%)	90(46.39%)	42(21.65%)	3.58±0.83	4.12±0.69	<0.001*
**Voice control technique **
	0(0%)	1(0.52%)	11(5.67%)	64(32.99%)	91(46.91%)	27(13.92%)	3.46±0.81	3.91±0.71	<0.001*
**Distraction technique**
	2(1.03%)	14(7.22%)	46(23.71%)	76(39.18%)	46(23.71%)	10(5.15%)	2.89±1.09	2.96±0.96	0.606
**Modeling technique**						
	2(1.03%)	4(2.06%)	17(8.76%)	69(35.57%)	74(38.14%)	28(14.43%)	3.45±1.02	3.56±0.93	0.419
**Hand-over-mouth technique **					
	3(1.55%)	15(7.73%)	52(26.8%)	72(37.11%)	38(19.59%)	14(7.22%)	2.72±1.15	3.03±0.99	0.048*

### Demographics

Total number of 101 general dentists and 93 pedodontists were included in study. The mean age of the participants was 37.93±9.26 years (range 23-64 years). The mean work experience of the participants was 11.54±9.51 years (0-42 years). Table-1 presents the demographic variables of the participants. Most respondents (81.4%) reported that they had performed dental treatment for children in presence of parents, and 71.6% believed that parents were responsible for stress of dental clinicians. The Chi-square test showed a significant difference between males and females in provision of dental care for children under 12 years (96.3% of females versus 84.8% of males; P=0.023). The difference between males and females was not significant in ratio of pedodontists/general dentists (P=0.181), conduction of dental treatment in presence of parents (P=0.337), or main factor causing stress in clinicians (P=0.360). The difference between married and single participants was not significant in provision of dental care for children under 12 years (P=0.750), conduction of dental treatment in presence of parents (P=0.846), or the main factor causing stress in clinicians (P=0.311). However, the difference in ratio of pedodontists/general dentists was significant between married and single participants (P=0.032) such that 53.4% of pedodontists and 46.6% of general dentists were married.

Table-2 compares the pedodontists and general dentists regarding provision of dental care for children under 12 years, conduction of dental treatment in presence of parents, and the main factor causing stress in clinicians. The Chi-square test showed significant differences between the two groups in provision of dental care for children under 12 years (P=0.001) and conduction of dental treatment in presence of parents (P<0.001), such that a significantly higher percentage of pedodontists had performed dental treatment for children under 12 years and in presence of parents, compared with general dentists.

### Trait Stress

Table-3 presents the frequency distribution of responses of the two groups to questions in the trait stress domain of the questionnaire. Of all, 32% (n=62) stated that they would not prefer pediatric patients if they had a choice, 38.7% reported never experiencing stress during clinical dental examination of pediatric patients, and 43.4% reported stress during or even after completion of treatment of a child. Independent t-test indicated significantly lower stress score of pedodontists than general dentists in all items (P<0.05) except for stress during oral clinical examination of children and stress during dental procedures in presence of parents (P>0.05).

Assessment of the total trait stress score of pedodontists and general dentists with the average value (20) showed that the mean total trait stress score (11.41±5.53) in both groups (P<0.001) and also separately in pedodontists (8.48±4.34, P<0.001) and general dentists (14.10±5.14, P<0.001) was significantly lower than the average value (20). In other words, both groups had low trait stress in management of pediatric dental patients.

Independent t-test showed that the mean trait stress score of general dentists was significantly higher than that of pedodontists (14.10±5.14 vs. 8.48±4.34, P<0.001).

### Functional Stress

Table-4 presents the frequency distribution of responses of the two groups to questions in the functional stress domain of the questionnaire. As shown, the highest functional stress score was related to stress experienced during an inferior alveolar nerve (IAN) Block in an anxious child (1.69) while the lowest functional stress score was related to cavity preparation for an amalgam restoration of a mandibular tooth (0.85). Independent t-test showed that in all procedures, pedodontists experienced significantly lower level of stress than general dentists (P<0.05).

Independent t-test compared the functional stress score of male and female participants and revealed that the mean stress score of male participants was lower than that of female participants during administration of an IAN block (mean score of 1.61±1.04 in females vs. 1.18±1.10 in males, P=0.033). No other significant differences were found between males and females (P>0.05).

The mean functional stress score was 16.84±11.52 (range 0 to 44) in total, 8.65±5.92 (range 0 to 25) in the mandible, and 8.18±5.84 (range 0 to 22) in the maxilla. Independent t-test showed no significant difference in the mean functional stress score in total, or separately in the mandible and maxilla between males and females (P>0.05). However, the mean functional stress scores in total (P<0.001), and separately in the mandible (P<0.001) and maxilla (P<0.001) were significantly lower in pedodontists than general dentists. The mean functional stress score in total (16.84) was significantly lower than the average level (35) (P<0.001). Also, the mean functional stress score in total in pedodontists (13.50) and general dentists (19.91) was lower than the average level (P<0.001). The mean functional stress score of the mandible (8.65) and maxilla (8.18) was also significantly lower than the average level (17.5) (P<0.001). The mean functional stress score of the mandible in pedodontists (6.90) and general dentists (10.27) was also lower than the average level (17.5) (P<0.001). The mean functional stress score of the maxilla in pedodontists (6.60) and general dentists (9.63) was lower than the average level (17.5) as well (P<0.001).

### Attitude

Table-5 presents the frequency distribution of responses of the two groups to questions in the attitude domain of the questionnaire. As shown, the Tell-Show-Do technique acquired the highest score (3.84) while the hand-over-mouth technique acquired the lowest score (1.21).

Independent t-test showed that the mean attitude score of pedodontists was significantly higher than that of general dentists regarding the role of knowledge about pediatric psychological theories in reduction of children’s stress during dental procedures (P=0.030), optimal efficacy of behavioral control techniques in stress management of children (P=0.001), efficacy of the tell-show-do technique (P<0.001), and efficacy of the positive reinforcement technique (P<0.001).

No significant difference was found between males and females regarding their attitude towards different parameters (P>0.05) as shown by independent t-test. Also, the mean attitude score of behavioral control (P=0.808) and the mean attitude score of application of behavioral control techniques (P=0.591) were not significantly different between males and females. However, the mean attitude score of pedodontists was significantly higher than that of general dentists regarding behavioral control (P=0.004) and application of behavioral control techniques (P=0.019). The mean behavioral control attitude score (25.63) (P<0.001) was significantly higher than the average value of 20, and the mean attitude score for application of behavioral control techniques (18.05) (P<0.001) was significantly higher than the average value of 15. The mean behavioral control attitude score of pedodontists (26.63) and general dentists (24.73) was significantly higher than the average value of 20. The mean attitude score for the application of behavioral control techniques in pedodontists (18.68) and general dentists (17.46) was significantly higher than the average value of 15 as well.

## Discussion

This study assessed the stress level of general dentists and pedodontists in dental treatment of pediatric patients. The results showed significant differences between general dentists and pedodontists regarding conduction of dental treatments for children under 12 years, treatment in presence of parents, and gender, such that 100% of pedodontists versus 89.1% of general dentists reported conduction of dental treatment for children under 12 years of age. Also, 91.4% of pedodontists versus 72.3% of general dentists reported conduction of dental procedures in presence of parents. Furthermore, 96.3% of females versus 84.8% of males reported conduction of dental treatment for children under 12 years of age. The trait stress score in general, and separately in the two groups, was lower than the average value, indicating low trait stress of dental clinicians.

The mean functional stress score in total, and separately in the maxilla and mandible, was also lower than the average value. Also, the function stress for both jaws was lower in pedodontists than general dentists. Trait stress and functional stress of both jaws were significantly higher in general dentists than pedodontists, which was in line with the results of Anabuki et al, [11] who reported low stress level of pedodontists. Also, Kim and Lee [14] indicated lower occupational stress of pedodontists than general dentists.

In the present study, the participants reported the highest level of stress during administration of an IAN block for anxious children, and the lowest stress during cavity preparation for an amalgam restoration of a mandibular tooth. In all procedures, pedodontists reported lower stress level than general dentists. In all dental procedures except for anesthetic injection and pulpotomy, the stress level was higher for procedures in the maxilla than mandible, which can be due to differences in vision (indirect versus direct). Also, IAN block is associated with higher stress level for clinicians compared with infiltration anesthesia in the maxilla due to different anatomy of the mandibular nerve, the need for sufficiently maintaining the mouth open, and masking the child’s vision. Farokh-Gisour and Hatamvand [10] reported that administration of an IAN block for an anxious child was the most stressful procedure in the department of pediatric dentistry. Similarly, Davidovich et al. [7] demonstrated that anesthetic injection for an anxious child was the most stressful procedure for general dentists and pedodontists.

Rasmussen et al. [10] showed that administration of an IAN block for preschool children was the most stressful method of pain control. Also, Azher et al. [12] discussed that anesthetic injection was the most stressful and most difficult procedure for children. Farokh-Gisour and Hatamvand [10] indicated that cavity preparation for an amalgam restoration of a mandibular tooth was the least stressful procedure. Their results were in line with the present findings.In the present study, the stress score of males in administration of an IAN block was lower than that of females. Azher et al. [12] reported higher stress level in females than males, which was in line with the present results. Difference in stress level of males and females can be due to their different psychological state and emotions. Also, men less commonly express their feelings, and higher stress level in women may be due to their reaction to stressful situations and their delicate nature [11].

Similarly, Samkhanian and Eftekhari [15] reported higher mean level of stress and anxiety in female dentists than male dentists; however, the differences did not reach statistical significance. Davidovich et al, [7] however, found no significant difference in stress score of male and female dentists for any treatment procedure, which was different from the present findings. This difference may be due to variations in study populations and cultures.

In the present study, the highest efficacy score was given to the tell-show-do technique and the lowest score was allocated to the hand-over-mouth technique. Also, the attitude score of pedodontists towards the significance of knowledge about pediatric psychological theories, efficacy of behavioral control techniques in stress management, and efficacy of the tell-show-do, positive reinforcement, and modeling techniques was significantly higher than that of general dentists, which may be due to greater experience and higher self-esteem of pedodontists than general dentists. Moreover, the attitude score of pedodontists towards the efficacy of behavioral control techniques and their application was higher than that of general dentists. Crossley and Joshi [16] demonstrated that the tell-show-do technique was the most accepted and the hand-over-mouth technique was the least accepted technique. Nazzal et al. [17] discussed that the tell-show-do and positive reinforcement techniques were the most commonly used techniques by pedodontists while the hand-over-mouth technique was the least commonly used technique.

Future studies with a larger sample size are required to compare the stress level of dental students with general dentists and pedodontists.

## Conclusion

The stress level of both pedodontists and general dentists was lower than the average level. The stress level of pedodontists was generally lower than that of general dentists in all procedures. Administration of an IAN block for an anxious child was the most stressful procedure for both general dentists and pedodontists, and the tell-show-do technique was the most effective behavioral control measure.

## Conflict of Interest

The authors declare no competing interests.
